# Potential Human Adaptation Mutation of Influenza A(H5N1) Virus, Canada

**DOI:** 10.3201/eid2009.140240

**Published:** 2014-09

**Authors:** Sebastian Maurer-Stroh, Yan Li, Nathalie Bastien, Vithiagaran Gunalan, Raphael Tze Chuen Lee, Frank Eisenhaber, Tim F. Booth

**Affiliations:** Agency for Science, Technology and Research, Singapore (S. Maurer-Stroh, V. Gunalan, R.T.C. Lee, F. Eisenhaber);; Ministry of Health, Singapore (S. Maurer-Stroh);; Nanyang Technological University, Singapore (S. Maurer-Stroh, F. Eisenhaber);; Public Health Agency of Canada, Winnipeg, Manitoba, Canada (Y. Li, N. Bastien, T.F. Booth);; University of Manitoba, Winnipeg (Y. Li); and National University of Singapore, Singapore (F. Eisenhaber)

**Keywords:** viruses, influenza, A(H5N1), Canada

**To the Editor:** In December 2013, influenza associated with pandemic influenza A H5N1 was reported in Canada in a patient who had traveled to China; the patient died in January 2014. This case leaves unanswered questions.

In the absence of direct poultry contact by the patient, the possible route of transmission and infection, often influenced by receptor-binding properties of the virus, requires special attention. The full genome and phylogenetic analysis by Pabbaraju et al. (*1*) provides a summary of what can typically be deduced from the sequence. The authors also mention 2 novel mutations, R189K and G221R, in the hemagglutinin (HA) protein (R193K and G225R in H3 numbering, used hereafter). When mapped to the H5 HA protein structure by using FluSurver in GISAID (http://www.gisaid.org, http://flusurver.bii.a-star.edu.sg), both mutations are found in the immediate receptor-binding pocket, and G225R has been known to change specificity of an H3N2 virus toward human erythrocytes (*2*). The same position is also known for receptor recognition changes in the 2009 pandemic H1N1 virus (mutations D222G, D225G, or D239G in different numberings). Besides A/Alberta/01/2014 (clade 2.3.2.1c), the mutation G225R has been found in 3 other H5N1 sequences: A/duck/Hunan/15/2004 (clade 2.3.3), A/chicken/Xinjiang/53/2005, and A/chicken/Xinjiang/27/2006 (both clade 7, all lineage assignments made with LABEL, http://label.phiresearchlab.org/). Although few G225R mutations were found, they were all found in avian hosts, indicating that the mutation can occur sporadically and avian-like receptor-binding properties may not be fully abolished by G225R.

In the absence of glycan-binding data or crystal structures, which take longer to deduce, computational structural modeling is an efficient and safe alternative for fast preliminary assessment of these mutations in their natural structural context of H5N1 binding pockets. We have shown (*3*) that a method using the classical AMBER03 molecular mechanics force field (*4*) with an implicit solvation model in combination with short molecular dynamics simulations in YASARA (*5*) can reproduce relative preference for human-like α2,6-linked versus avian-like α2,3-linked sialic acid receptors. The interaction energies of all atoms in a system are described and combined with distance-dependent functions for different interaction types, including bonds, various angles, van der Waals, electrostatics, and solvent, which leads to consideration of the concerted effects of all residues in the binding pocket. By using this energy function, short molecular dynamics simulations enable all atoms to move for specified intervals within the constraints of their interactions. These simulations are used to minimize and finally predict the energies of the wildtype and mutant HA proteins for their ligand-bound and unbound states considering their respective ligands (see Methods section of [*3*] for details). 

In this study, we further tested the computational structural model on mutations with known effect on receptor-binding properties (*2*,*3*,*6*–*9*) in H5N1 context based on recently resolved crystal structures and the respective ligand complexes (*9*). We limited this selection to mutations in the immediate vicinity of the crystallized receptor analog because the method should be most accurate for this scenario. The results showed that the binding preference of known mutations could be predicted at least qualitatively. Next, we tested the additional mutations found in A/Alberta/01/2014(H5N1). Our results (Technical Appendix Figure) suggest that G225R could incur a relative predicted increase in binding to the human-like receptors. Although the quantitative accuracy of computational methods in this regard is limited, the predicted numerical value suggests a possible similar extent of the effect to that of the well-known Q226L mutation. It should also be noted that the predicted increase in binding to human-like receptors does not necessarily imply a concomitant loss of avian receptor binding. The role of R193K is less clear with a slight predicted tendency of favoring avian-like receptors. These preliminary findings highlight the necessity of verifying not only the receptor-binding properties of this virus through experimentation, but given the predicted increased preference for human receptors, also verifying potential roles in altered mammalian transmissibility.

These receptor-binding pocket mutations of the virus were not seen in the most closely related Asian H5N1 sequences of clade 2.3.2.1c (*1*), and no human contacts were known to be affected. From the epidemiologic perspective of this isolated human case, it is possible that this variant arose in the patient after initial infection and contributed to prolonged and severe infection and to the more unusual spread to brain tissue. If more avian strains with G225R mutations are found, the example of Q226L in H7N9 indicates that relative receptor-binding changes alone do not necessarily imply immediate mammalian transmissibility (*10*). It should also be noted that G225R was not among the mutations identified by recent controversial mammalian adaptation studies, (*7*,*8*) indicating that there may be more H5N1 host specificity markers than have been identified. Consequently, the functional roles of G225R in avian influenza should be further analyzed by conducting secure experiments and, pending verification, checking closely for its potential as an avian influenza host specificity marker.

Technical Appendix Computational structure modeling and specificity of human receptor binding. A new HA receptor-binding pocket mutation in the influenza A/Alberta/01/2014(H5N1) virus is predicted by computational structural modeling that will alter specificity toward a relative increase in human receptor binding.
