# Supplementary material for: Potential Human Adaptation Mutation of Influenza A(H5N1) Virus, Canada
Source: Emerg Infect Dis. 2014 Sep;20(9):1580–2. doi: 10.3201/eid2009.140240 (PMC4178386; doi:10.3201/eid2009.140240)
Supplement: Technical Appendix — Computational structure modeling and specificity of human receptor binding. A new HA receptor-binding pocket mutation in the influenza A/Alberta/01/2014(H5N1) virus is predicted by computational structural modeling that will alter specificity toward a relative increase in human receptor binding. [file 14-0240-Techapp-s1.pdf]

DOI: http://dx.doi.org/10.3201/eid2009.140240

Potential Human Adaptation Mutation of Influenza A(H5N1) Virus, Canada

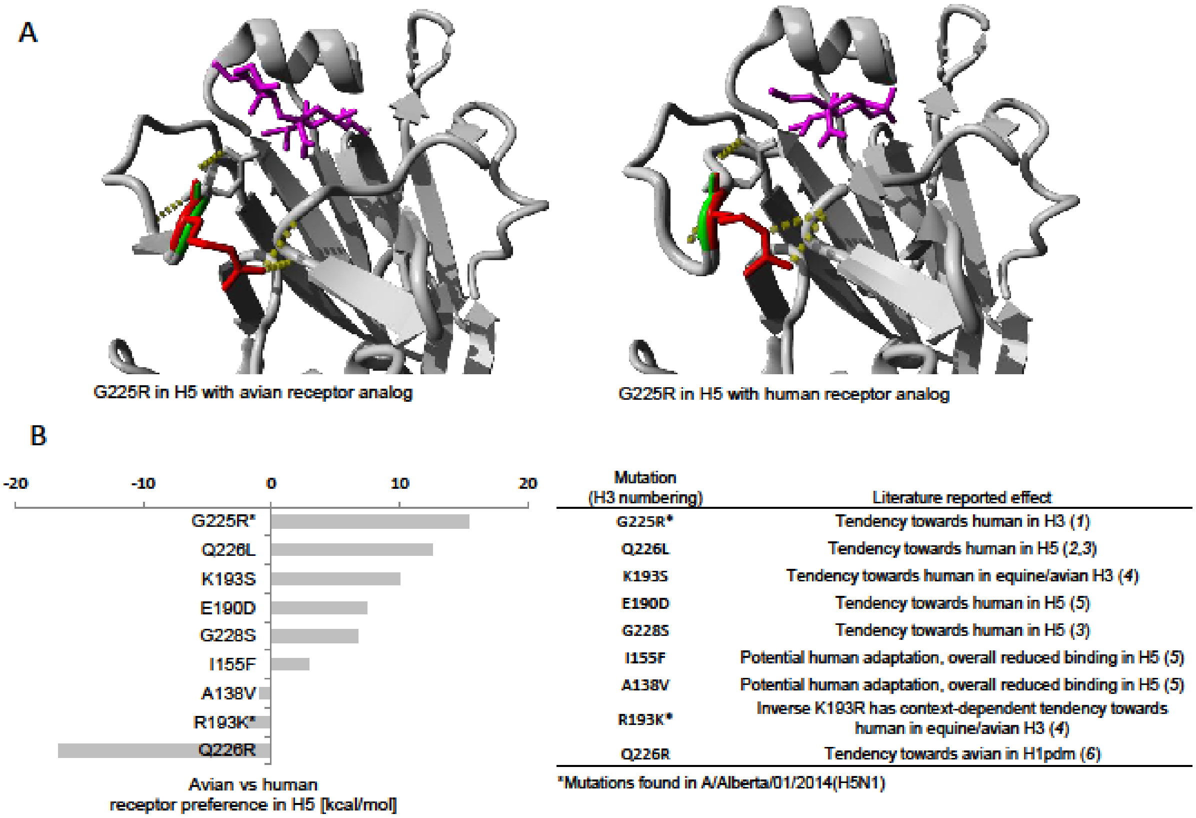

Mutation (H3 numbering)	Literature reported effect
G225R*	Tendency towards human in H3 (*1*)
Q226L	Tendency towards human in H5 (*2,3*)
K193S	Tendency towards human in equine/avian H3 (*4*)
E190D	Tendency towards human in H5 (*5*)
G228S	Tendency towards human in H5 (*3*)
I155F	Potential human adaptation, overall reduced binding in H5 (*5*)
A138V	Potential human adaptation, overall reduced binding in H5 (*5*)
R193K*	Inverse K193R has context-dependent tendency towards human in equine/avian H3 (*4*)
Q226R	Tendency towards avian in H1pdm (*6*)

*Mutations found in A/Alberta/01/2014(H5N1)

Technical Appendix Figure. G225R, a new HA receptor binding pocket mutation in A/Alberta/01/2014(H5N1) is predicted by A) computational structural modeling that will B) alter specificity toward a relative increase in human receptor binding. A) Modeled hemagglutinin complex with the avian-like receptor that is based on PDB:3zp0 on the left and the human-like receptor based on PDB:3zp1 on the right side. G225R facilitates creation of new hydrogen bonds to the opposite disulfide-bridge stabilized scaffold (≈S137), thereby slightly moving the 225 loop holding critical residue Q226, which is in direct contact with the ligand and appears slightly altered in the model of the complex with the human-like ligand. This provides a hypothetical mechanism for the binding changes. Colors indicate properties as follows: green: Wildtype G225; red: Mutant 225R; magenta: Receptor analog; yellow: H-bonds. B) Predicted tendencies of receptor preference changes on the left; positive values indicate increased

preference for human and negative values for avian receptors, respectively. Exact values may not be accurate but qualitative tendencies have been shown to be reproducible compared to experiments. The method was also applied to several binding pocket mutations with known effect reported in the literature and the predictions match the experimental observations summarized on the right side.

References

1. Martín J, Wharton SA, Lin YP, Takemoto DK, Skehel JJ, Wiley DC, et al. Studies of the binding properties of influenza hemagglutinin receptor-site mutants. Virology. 1998;241:101–11. PubMed http://dx.doi.org/10.1006/viro.1997.8958

2. Imai M, Watanabe T, Hatta M, Das SC, Ozawa M, Shinya K, et al. Experimental adaptation of an influenza H5 HA confers respiratory droplet transmission to a reassortant H5 HA/H1N1 virus in ferrets. Nature. 2012;486:420–8. PubMed

3. Herfst S, Schrauwen EJA, Linster M, Chutinimitkul S, de Wit E, Munster VJ, et al. Airborne transmission of influenza A/H5N1 virus between ferrets. Science. 2012;336:1534–41. PubMed http://dx.doi.org/10.1126/science.1213362

4. Medeiros R, Naffakh N, Manuguerra J-C, van der Werf S. Binding of the hemagglutinin from human or equine influenza H3 viruses to the receptor is altered by substitutions at residue 193. Arch Virol. 2004;149:1663–71. PubMed http://dx.doi.org/10.1007/s00705-003-0287-2

5. Crusat M, Liu J, Palma AS, Childs RA, Liu Y, Wharton SA, et al. Changes in the hemagglutinin of H5N1 viruses during human infection–influence on receptor binding. Virology. 2013;447:326–37. PubMed http://dx.doi.org/10.1016/j.virol.2013.08.010

6. Guarnaccia T, Carolan LA, Maurer-Stroh S, Lee RTC, Job E, Reading PC, et al. Antigenic drift of the pandemic 2009 A(H1N1) influenza virus in A ferret model. PLoS Pathog. 2013;9:e1003354. PubMed http://dx.doi.org/10.1371/journal.ppat.1003354
